# Reviews

**Published:** 1881-10

**Authors:** 


### Reviews.

**A Medical Formulary,** based on the U. S. and British Pharmacopœia, together with numerous French, German, and unofficinal preparations. By Laurence Johnson, A. M., M. D., Lecturer on Medical Botany Medical Department University of New York. New York: Wm. Wood & Co.

When we say that this work is the least valuable of the series published this year, it is only because the other volumes of "Wood's Library for 1881" have been so valuable that no mere collection of formulæ, however good in itself, can be ranked with them.

**A Treatise on Bright's Disease and Diabetes, with special reference to Pathology and Therapeutics.** By JAMES TYSON, A. M., M. D., Prof. of General Pathology and Morbid Anatomy in the University of Pennsylvania, &c. With illustrations. Philadelphia: Lindsay & Blakiston. 1881.

The opening chapters of this work are devoted to a study of the anatomy of the kidneys as seen by the naked eye and with the aid of the microscope, both healthy and pathological. These chapters show a close and extended research, and contain much original matter. In the following portions albuminuria and casts receive the attention which these important subjects deserve. Under the head of "Casts" the author makes the following concluding observations:

"1. Hyaline casts are found in all forms of Bright's disease, as well as in temporary congestions of the kidney, active or passive.

"2. Epithelial casts are found in acute, subacute and chronic parenchymatous nephritis. In the latter two forms the cells are generally degenerated and fragmentary.

"3. Blood casts are found in acute parenchymatous nephritis, and where hæmorrhages have occurred in the kidneys.

"4. Pale granular casts are found in interstitial nephritis (contracted kidney) and chronic parenchymatous nephritis (large white kidney).

"5. Dark granular casts are found in parenchymatous nephritis, acute and chronic, and rarely in interstitial nephritis.

"6. Waxy casts are found only in chronic Bright's disease, and attend either of the three principal forms.

"7. Oily casts are found in subacute and chronic forms of Bright's disease, and may attend any of the three principal forms, but are most numerous in chronic parenchymatous nephritis.

"8. Free fatty cells and free oil drops are found in chronic parenchymatous nephritis.

"9. The form of fatty cell known as the compound granular cell is found in acute and chronic parenchymatous nephritis."

The divisions of Bright's disease adopted are into: I, Acute Bright's disease, acute parenchymatous nephritis; II, Chronic

Bright's disease, including (1) chronic parenchymatous nephritis, (2) lardaceous disease, (3) interstitial nephritis; adding chapters upon cyanotic induration and suppurative interstitial nephritis.

We are more than usually pleased with this portion of the work. The section devoted to Diabetes impresses us as a very complete account of what is known and what is believed regarding this as yet somewhat obscure disease. The experimental investigations hitherto made, as well as the physiological and pathological bearings of the disease, are reviewed in detail. In other respects, including treatment, the work embodies the most recent views.

---

**A System of Surgery, Theoretical and Practical; in Treatises by various Authors.** Edited by T. HOLMES, Surgeon and Lecturer on Surgery at St. George's Hospital. First American, from second English edition; thoroughly revised and much enlarged. By JOHN H. PACKARD, M. D., Surgeon to the Episcopal and St. Joseph's Hospital, Philadelphia. Assisted by a large corps of the most eminent American Surgeons. In three volumes. Philadelphia: Henry C. Lea's Sons & Co. 1881.

Since the appearance of Ziemssen's Cyclopædia of Medicine, the profession has felt the want of a similar work, embracing the science of surgery in all its details. Surgery has in the last twenty years made such rapid progress, that it is next to impossible for one mind to be equally well at home in all the different branches, as to write a work in which full justice would be given to every subject. Holmes' Surgery, written by the best-known English surgeons, has been long known and appreciated as such a work, and an edition of this work by well-known American surgeons cannot fail to be hailed with pleasure by the profession. Vol. I, which has just made its appearance, treats of General Pathology, Morbid Processes (Abscesses, Sinus and Fistula, Gangrene and Ulcers); Injuries in General, Complications of Injuries, and Injuries of Regions, and the text of the last English edition has been reproduced, unaltered and unabridged, the comments of the American editors having been simply interpolated. Each volume will have a copious index at the end, and the work will be concluded with a more general one, so that the labor of reference to any particular point will be reduced to a minimum. For the busy practitioner, who has discovered that the purchase of Ziemssen's Cyclopædia was a useless expense, till a general index was provided and *extras* paid for, this will be appreciated. The revision has been carefully made by the American corps of editors, and the work, as now presented, gives a complete discussion of the most recent views, regarding the various departments of surgery.

---

**The Mother's Guide in the Management and Feeding of Infants.** By JOHN M. KEATING, M. D., Lecturer on Diseases of Children at the University of Pennsylvania, etc. Philadelphia: Henry C. Lea's Sons & Co. 1881 Buffalo: Theodore Butler.

We have examined this little work with care, and have been repaid for the time and labor thus bestowed. It is a very useful contribution to the literature of the subject of which it treats, useful to the mother in the information it conveys, and to the practitioner as a hand-book for distribution among his patients. We cordially commend it to the profession.

---

**Anatomical Studies upon the Brains of Criminals.** A contribution to Anthropology, Medicine, Jurisprudence and Psychology. By MORITZ BENEDIKT, Professor at Vienna. Translated from the German by E. P. FOWLER, M. D., New York. New York: Wm. Wood & Co., 27 Great Jones St. 1881.

The translator offers an explanation for his labor in placing this work before the English-reading public, that it is a gratuitous contribution towards establishing a scientific basis for the prevention of crime. The anatomical construction and physiological development of the brain are at the base of the system it is the design of this work to establish. In the introduction the author takes up the normal brain and gives a clear exposition of its principal anatomical structures, after which he illustrates the subject by an examination of the brain of the most noted criminals. It will be seen, therefore, that from these premises the author endeavors to prove crime as a psychological act of the criminal, and the criminal is therefore the first object of study.

The work is certainly a valuable one. It unfolds a line of thought well worthy of study by the profession, and especially by the authorities to whom are entrusted the prevention of vice.

---

**A Manual of Histology.** Edited and prepared by THOMAS E. SATTERWAITE, M. D., of New York, President of the New York Pathological Society, &c., in association with Drs. THOMAS DWIGHT, J. COLLINS WARREN, and others. With one hundred and ninety-eight illustrations. New York: William Wood & Co. 1881.

This work is intended for the student and practitioner. Pathology and pathological histology receive notice in a way to call attention to the practical application of the facts of histology, which might otherwise appear valueless as matter of information. The author in his preface speaks of "the goodly number of medical men who are either engaged in teaching histology or in studying some special branch of it." This is rather a questionable statement. The small number of medical men engaged in original research in this country is properly a subject of reproach. The proportion, as compared with that in European countries, is insignificant. The excuse of former years, that this country is too new, can no longer be considered a satisfactory answer. It ought not to be many years before this country shall be able to point to the peers of such men as Stricker, Rindfleisch and Ranvier.

Again the author remarks: "In some respects the object sought for has not been wholly attained, as, for example, in the effort to separate purely human histology from the comparative." It may well be asked whether this separation of human from comparative histology is advisable. We are more inclined to agree with the views of the speaker who delivered the opening address of the section of Pathology at the recent International Medical Congress. In the course of the address the speaker calls attention to the great benefit to be derived from a less limited scope of research. The fact that different animals afford peculiar advantages in the study of various histological and pathological subjects is too well known to require affirmative argument.

In reviewing the book we are inclined to think favorably of its filling "the obvious gap" to which the writer refers. While original matter is not to be expected in a work of this nature, it yet gives a concise review of the histology of to-day, a practical tendency being apparent throughout the volume.

---

**Atlas of Gynæcology and Obstetrics.** Edited by Dr. A. MARTIN, Professor of Gynæcology in the University of Berlin. Containing 475 black and 37 colored illustrations from the original designs by Brigel, Virchow, Hyrtl, Kilian, Naegele, Schroeder, etc. Supplemented by numerous illustrations from J. P. Maygreir's "Nouvelles Demonstrations d'Accouchements." Price, $1.00 for each part. Cincinnati : A. E. Wilde & Co., publishers.

In the prospectus, the publishers announce that the "Atlas of Gynæcology and Obstetrics" will be published in fifteen parts, each part containing four elegant plates and four pages of explanatory text. We have received parts I, II, III, and IV. They are issued in the same size and style of the "Anatomical Atlas," by the same publishers, which we have had occasion to commend to the profession in previous numbers of the JOURNAL, and will constitute an equally valuable addition to the library of the practitioner. The illustrations are of sufficient size to portray clearly the subject; and in artistic finish the "Atlas" will be far superior to any work yet published for the moderate cost at which the enterprising publishers are able to offer it. A careful examination of the plates and the design of the work impress us favorably, and we commend it to the profession.

